# A Study of the Gas-Stabilized Arc as an Emission Source for the Measurement of Oscillator Strengths. Determination of Some Relative *gf*-Values for Fe I*

**DOI:** 10.6028/jres.067A.053

**Published:** 1963-12-01

**Authors:** Marvin Margoshes, Bourdon F. Scribner

## Abstract

A study has been made of the application of the gas-stabilized arc source to the determination of oscillator strengths in atomic spectra. In this source, a solution of salts of one or more elements may be injected and the elements excited directly in an arc plasma with steady emission of spectral intensities. The source can be taken to be characterized by local thermal equilibrium, but with a large radial temperature gradient. An experiment indicates that when two elements are introduced into the arc in a solution, the relative concentrations of the elements in the discharge are the same as in the solution within the probable experimental error. This experiment indicates one way that the arc may be used for the determination of absolute oscillator strengths. New measurements are reported of the *gf*-values of 105 lines of Fe I between 2900 and 4150 Å, and the new values are compared with the results of earlier measurements.

## 1. Introduction

The gas-stabilized arc source was developed initially for the spectrographic analysis of solutions [1].^1^ The source is an arc between a graphite anode and a thoriated tungsten cathode. The arc is stabilized in position by a stream of argon, and samples are introduced into the arc column as solutions in the form of a fine spray. In the course of studies of the properties of this discharge, it became apparent that the stability of emission intensities and the ease of introduction of the elements in a controlled manner might make the source applicable to the measurement of oscillator strengths in atomic spectra.

The intensity, *I_ij_*, of a line resulting from transitions between an upper energy level, *i*, and a lower energy level, *j*, can be related to the transition probability, *A_ij_*, when the source is at thermal equilibrium and when self-absorption and induced transitions can be neglected:

$$I_{ij}=D(N/u)hv_{ij}g_{i}A_{ij}\;\exp(-E_{i}/kT),$$ (1)

where *D* is the depth of the emitting vapor, *N* is the number of atoms or ions per unit volume, *h* is Planck’s constant, *g_i_* is the statistical weight of the excited state, *E_i_* is the energy of the upper level of the transition, *k* is Boltzmann’s constant, *T* is the absolute temperature, and *u* is the partition function, equal to Σ*_n_g_n_* exp (*‒E_n_/kT*). A similar equation may be writ ten in terms of the oscillator strength *f_ij_*:

$$I_{ij}=-D(N/u)(8\pi^{2}\epsilon^{2}hv_{ij}^{3}/mc^{3})g_{i}f_{ij}\exp(-E_{i}/kT),$$ (2)

where *ϵ* and *m* are the charge and mass of an electron, and *c* is the velocity of light. There is a simple relation between the oscillator strengths for emission and absorption, *g_i_f_ij_*= ‒*g_i_f_ji_*, while the relation between the emission and absorption transition probabilities is more complex.

If atomic oscillator strengths or transition probabilities are known, it is possible to derive considerable information from the study of the intensities of emission or absorption spectra. For example, rewriting eq (2) in logarithmic form, rearranging, and substituting *c/*λ for *v*, gives

$$\log(I_{ij}\lambda_{ij}^{3}/g_{i}f_{ij})=\log\;K-0.4343E_{i}/kT,$$ (3)

where *K* includes those constants of eq (2) not explicitly included in eq (3). If the emitting atomic vapor is in thermal equilibrium, a plot of the left-hand side of eq (3) versus *E_i_* will give a straight line with a slope equal to ‒0.4343/*kT*. In this way, the temperature of the atomic vapor may be determined. The temperature may also be measured from the intensity ratio of only two lines. For this purpose, eq (2) should be written in the form

$$I_{ij}/I_{mn}=(v_{ij}^{3}g_{i}f_{ij}/v_{mn}^{3}g_{m}f_{mn})\exp[(E_{m}-E_{i})/kT].$$ (4)

Equations similar to (6) and (4) can also be written in terms of the transition probabilities.

If the vapor is not in thermal equilibrium, the intensities of the emission lines can be used to determine the numbers of atoms in individual energy levels, and these data can provide some indication of the mechanisms of energy transfer. Transition probabilities and oscillator strengths are of extreme importance in astrophysics, where their knowledge makes it possible to obtain information from solar and stellar spectra, including chemical composition, that could not be obtained in any other way.

Oscillator strengths or transition probabilities may be measured from the absorption or emission spectra of atomic vapor having a uniform, known composition and temperature. The King furnace [2] can provide such a vapor suitable for absorption measurements. However, measurements with the King furnace normally provide information only on lines for which the lower energy level is at or near the ground state, since the temperatures attainable with this furnace are not high enough to give any significant concentration of atoms in the higher energy levels. Measurements of atomic transition probabilities and oscillator strengths have been made from emission spectra in flames and arcs. Flames can be made reasonably homogeneous in temperature and composition, but they are too cool to excite many atomic lines, and even fewer ion lines. Arcs are not usually homogeneous in composition and temperature, but if they are stable it may be possible to study their composition and temperature as a function of position in the arc column. The studies described here were designed to investigate the applicability of the gas-stabilized arc source to the measurement of oscillator strengths.

## 2. Description of the Source

**Figure 1:**
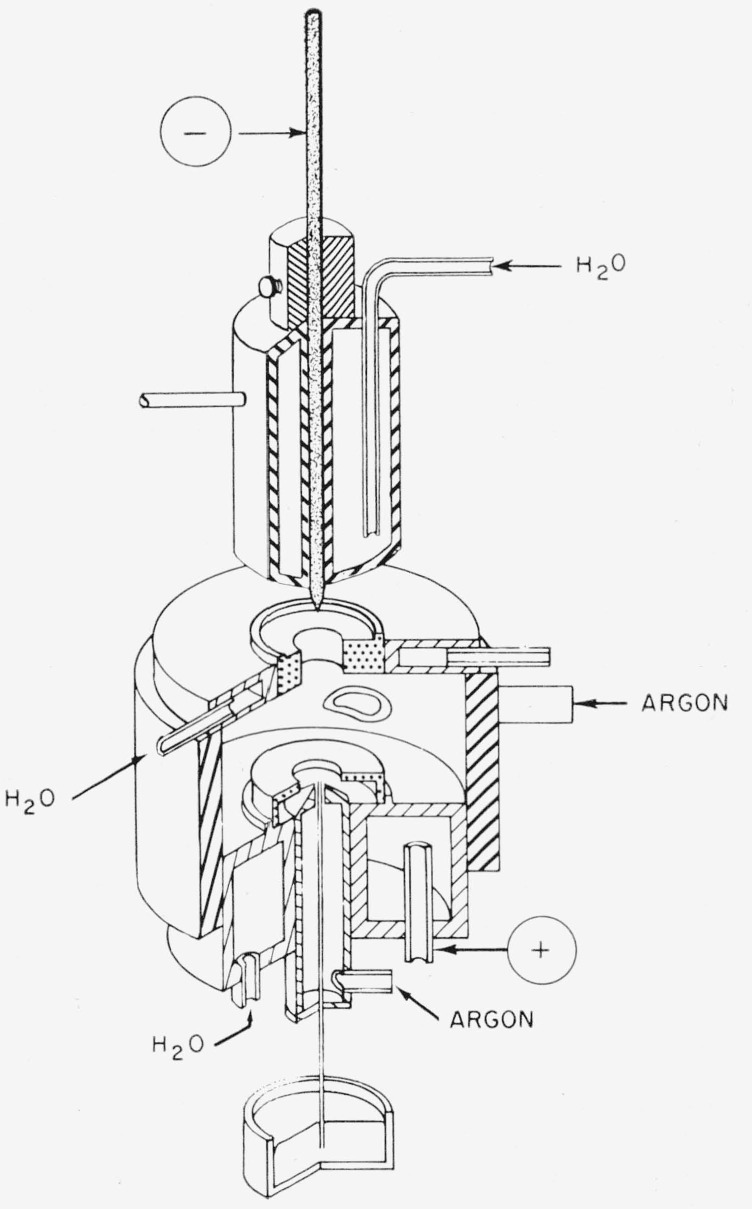
Schematic diagram of the gas-stabilized arc source.

A complete description of the source has been given previously [1], and only a summary is given here. Figure 1 is a schematic diagram of the gas- stabilized arc source. The anode is a graphite disk with an opening in the center. The atomizer is located just below the anode, and it is held in a water- cooled brass cylinder by a poly(tetrafluoroethylene) collar which keeps it electrically insulated from the discharge. The orifice is another graphite disk which is held in a water-cooled brass plate mounted on a Bakelite sleeve fitting snugly over the brass cylinder holding the anode and the atomizer. In this way the orifice is electrically insulated from the rest of the source so that it may take on the local potential of the arc. A stream of argon is introduced tangentially through the Bakelite sleeve to control the position of the arc. The cathode is a thoriated tungsten rod which is held vertically on the axis of the discharge by a water-cooled copper chamber.

The electrical power for the discharge may be provided by a d-c arc rectifier power supply or by d-c line power controlled by a ballast resistor. Either type of power supply is satisfactory, but an inductor in the ouput is necessary for stable operation.

When no solution is introduced, the arc burns quietly with very little fluctuation in position. When water or a solution is introduced, the arc becomes somewhat turbulent, but appears to remain stable in position. However, high-speed movies (4000 to 7000 frames/sec) show that, when water is sprayed in, the arc moves about in a random manner and only appears to be stable because the motion is extremely rapid.

The voltage drop between the electrodes is 40 v when no sample is introduced and rises to about 50 v when 0.2 ml/min of water is sprayed in. The arc current and voltage remain stable for extended periods without any adjustment of the power supply. The arc may be operated for 30 min or more at a current of 20 amp and a sample spray rate of 0.2 ml/min; the time of operation is limited by attack by water vapor on the hot tungsten cathode or, in some cases, the deposition of material from the solution onto this electrode. The graphite anode need be replaced only after a few hours of operation, and the orifice requires even less frequent replacement or cleaning.

The spectrum of the discharge shows lines of the elements in the solution and bands of OH and $N_{2}^{+}$, as well as lines of Ar and Ar^+^ and diffuse lines of H and N. The spectra of thorium and tungsten are strong at the cathode, but these lines decrease rapidly in intensity toward the orifice and are indistinguishable above background in the lower half of the gap. There is relatively strong continuous radiation from the arc, probably arising from recombination of argon ions and electrons.

The movement of the arc is rapid enough so that the measured emission intensities are quite constant, even with a short time constant in the recording system. When an emission line is recorded with a time constant of ½ sec, the fluctuations in intensity are about 5 percent, peak to peak [1]. Observations over a period of 30 to 40 min, with 30 sec integration of the signal, have shown [1] that the line intensities remain constant to within 1 or 2 percent.

## 3. Temperature of the Arc

Because of the rapid random motion of the arc, the distribution of temperatures in the discharge could be studied only if the exposure times were made very short. There has been no attempt to do this, and the data reported here represent averages in both space and time. For this reason, all temperatures given here should be considered to be effective excitation temperatures only.

Preliminary studies of the temperature of the arc have been reported previously [1]. These temperatures were determined from the relative intensities of four Cr II lines at 2860.93, 2862.57, 2870.44, and 2875.99 Å using relative transition probabilities given by Zagoryanskaya [3]. The measurements indicated a temperature of 8125 °K, and no systematic changes with temperature were found when the current and sample flow were varied.

The temperature of the arc has also been measured from the relative intensities of 41 lines of Ti II, 24 lines of Ti I, and 95 lines of Fe I. The measurements were based on *gf*-values given by King [4] for Ti II, and by Corliss and Bozman [5] for Ti I and Fe I. The experimental arrangement for these temperature measurements was the same as is described in section 5.1 of this paper.

**Figure 2:**
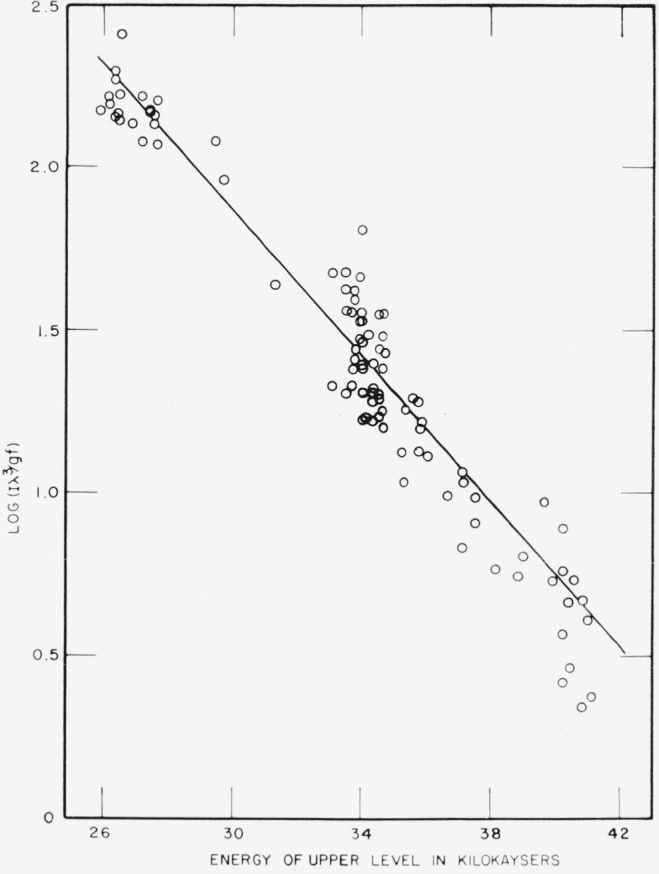
Plot of log (*Iλ*^3^/*gf*) versus energy of the upper state for lines of *Fe I* in gas-stabilized arc source, g*f*-Values taken from Corliss and Bozman [5], Straight line shows least squares fit to points.

For each of the thermometric species (Ti I, Fe I, Ti II) a plot was made of log (*I*λ^3^/*gf*) versus the energy of the upper state of the transition. Figure 2 shows the plot obtained in this way for Fe I. The slope of the best straight line for the data points was calculated by the method of least squares, and the temperature of the arc was found from *T*= ‒ 0.4343/*kb*, where *b* is the slope of the line. The temperatures found were: from Ti II, 7570 °K; from Ti I, 5540 °K; from Fe I, 5510 °K. The exposure for the measurement with Ti II was made with a solution containing both titanium and chromium; a temperature of 7680 °K was found in this exposure from the relative intensities of the four Cr II lines in the 2860 to 2875 Å region. The difference of 400° between the temperature obtained with the Cr II lines in previous experiments and in this exposure may be related to a small difference in the method of recording the spectra. The exposures indicating a temperature of 8125 °K were made with the source focused on the entrance slit of the spectrograph [1], while the exposure giving a temperature of 7680° K was made with the slit evenly illuminated with light passing through a mask on the source (see sec. 5.1). In the latter case, a wider area of the discharge was viewed and a lower temperature was found.

The plots of log (*I*λ^3^/*gf*) versus *E* were all linear within the precision of the data, which is consistent with an assumption of local thermal equilibrium (LTE). The fact that the measurements with Ti II and Cr II agree, as do those with Ti I and Fe I, is again consistent with LTE. However, this evidence does not establish that the arc is characterized by LTE. In this work, it was found that an effective excitation temperature could be determined for the arc. In addition, Kitaeva et al. [19] have found that an arc in argon is in LTE over the current range from 10 to 200 amp, which includes the current used in our work. Therefore, the arc was assumed to be characterized by LTE for the purpose of this study. If LTE is assumed, the disparity between the effective excitation temperatures obtained from the spectra of neutral atoms and of ions can be interpreted as indicating a large radial temperature gradiant in the arc.

## 4. Excitation of Mixed Solutions

Direct measurement of absolute transition probabilities with this source would be difficult because the concentration of an element in the discharge cannot easily be determined. Measured relative transition probabilities can be placed on an absolute scale if an absolute transition probability has been determined by some other method for at least one emission line. In the case of those elements for which no absolute transition probabilities are known, it may be possible to take advantage of the fact that two or more elements can be introduced into the source in known relative proportions. The assumption must then be made that the relative concentrations of the elements in the arc are the same as in the solutions being introduced, or else a correction must be made for differential volatilization or diffusion of the elements.

The possibility of performing measurements of this kind has been explored by introducing into the discharge a solution containing manganese and lead and measuring the intensity ratio for the line pair Mn 4031/Pb 2833 Å. Absolute *gf*-values for these lines have been determined in the same laboratory and by the same procedure [6, 7]. The expected intensity ratio was calculated assuming that the concentration ratio of the elements in the discharge was the same as in the solution. No attempt was made to correct for any self-absorption that might occur. Ionization in the arc was taken into account.

For a source characterized by local thermal equilibrium, ionization is described by the Saha equation:

$$N^{+}N_{\epsilon }/N{}^{\circ}=[{\left(2\pi mkT\right)}^{3/2}2u^{+}/h^{3}u{}^{\circ}]\;\exp\;(-V/kT),$$ (5)

where *N*^+^, *N_e_*, and *N°* are the concentrations of ions, electrons, and neutral atoms, *u^+^* and *u°* are the partition functions for the ion and the neutral atom at the temperature, *T*, of the discharge, *V* is the ionization energy of the neutral atom, and the other symbols have the same meaning as before. For the two elements Mn and Pb, eq (5) can be reduced to

$$\begin{array}{l} \left(N_{Mn}^{+}/N_{Mn}^{{}^{\circ}})/(N_{Pb}^{+}/N_{Pb}^{{}^{\circ}}\right)\\ \;=[(u_{\text{Mn}}^{+}/u_{\text{Mn}}^{{}^{\circ}})/(u_{\text{Pb}}^{+}/u_{\text{Pb}}^{{}^{\circ}})]\;\exp\;[(V_{\text{Pb}}-V_{\text{Mn}})/kT]. \end{array}$$ (6)

The first ionization energies of lead and manganese are 7.385 and 7.40 ev, so that the exponential term may be taken as equal to one. Substituting partition functions from Corliss and Bozman [5],

$$\begin{array}{l} \left(N_{\text{Mn}}^{+}/N_{\text{Mn}}^{{}^{\circ}})/(N_{\text{Pb}}^{+}/N_{\text{Pb}}^{{}^{\circ}}\right)\\ \;=(7.8/6.5)/(2.1/1.6)=0.92. \end{array}$$ (7)

To a reasonably good approximation, the degree of ionization should be the same for both elements, and the ratio of the concentrations of the neutral atoms should be about the same as the ratio of the total concentrations. Corliss [8] found that manganese was 29 percent ionized and lead was 31.5 percent ionized in the copper arc.

Equation (2) of section 1 may be rewritten for the intensity ratio of the Mn/Pb line pair:

$$\begin{array}{l} \left(I_{\text{Mn}}/I_{\text{Pb}}\right)\\ \;=(N_{\text{Mn}}^{{}^{\circ}}u_{\text{Pb}}^{{}^{\circ}}\lambda_{\text{Pb}}^{3}g_{\text{Mn}}f_{\text{Mn}}/N_{\text{Pb}}^{{}^{\circ}}u_{\text{Mn}}^{{}^{\circ}}\lambda_{\text{Mn}}^{3}g_{\text{Pb}}f_{\text{Pb}})\;\exp\;[(E_{\text{Pb}}-E_{\text{Mn}})/kT]. \end{array}$$ (8)

Because the spectra are of neutral atoms, the effective excitation temperature that should apply is the one obtained with the Fe I or Ti I lines. Assuming a temperature of 5510 °K, an intensity ratio of 1.61 was calculated for the ratio of the molar concentrations of lead and manganese in the experiment (*M*_Mn_/*M*_Pb_=2.72) The observed intensity ratios in two exposures were 2.17 and 2.03, average = 2.10. The ratio of calculated to observed relative intensities was 0.76.

The difference between the calculated and observed intensities might be due in part to demixing in the arc column [20], although it is to be hoped that the rapid passage of the gas through the excitation zone would limit the extent of demixing. The accuracies of the transition probabilities are also open to question. The precisions of the values, as determined by repeated measurements in the same laboratory and by the same method, are 8 to 10 percent [6, 7], but the accuracies of the values are not known.

These measurements cannot provide positive information on how accurately absolute oscillator strengths can be determined through the introduction of mixed solutions into the gas-stabilized arc source. However, the agreement between the observed and calculated intensity ratios in this one experiment is good enough to indicate that the technique will be of value.

## 5. Measurement of *gf*-Values for Fe I

As a part of this study, new measurements have been made of the oscillator strengths of 105 lines in the first spectrum of iron. Relative *gf*-values for these lines were determined by King and King [9] from furnace absorption measurements. Relative *gf*-values for a few of the lines were redetermined by Sobolev [10] from the spectrum of an arc between copper electrodes containing a small percentage of iron. More recent measurements of transition probabilities for most of these lines were made by Crosswhite [11] with a number of emission and absorption sources. Finally, Corliss and Bozman [5] have tabulated *gf*-values for most of these lines, based on measurements of spectra from an arc between copper electrodes containing 0.1 atomic percent of iron. The data of Cross white and those of Corliss and Bozman are relative values which have been placed on an absolute scale by means of conversion factors derived from absolute measurements.

Sobolev observed that his *gf*-values did not agree with those of King and King, and he ascribed the difference to a lack of thermal equilibrium in the King furnace. Crosswhite found a systematic difference between his measurements and those of King and King; when the two sets of data are placed on the same scale at longer wavelengths, the *gf*-values determined by the Kings are low by about a factor of 10 compared to Crosswhite’s values at shorter wavelengths. Crosswhite ascribed the wavelength-dependent difference to scattered light in the spectrograph used by the Kings.

### 5.1 Experimental Procedure

For measurements of the *gf*-values of Fe I, the source was operated at 20 amp and equipped with a mask arranged to pass light from a 1 mm-wide vertical cross-section through the axis of the arc in the lower half of the gap between the orifice and the cathode. The spectra were photographed in the first order of a 21-ft Wadsworth mount grating spectrograph with a 15,000 line/in. grating. The 30-*μ* entrance slit of the spectrograph was evenly illuminated, and four steps of a 1:2 step sector were employed to permit photometry of lines having widely different intensities. Two 10-in. SA-1 plates were used for each set of exposures in order to include the region from 2900 A to the long wavelength limit of the emulsion (about 4150 Å). The exposure time was selected to give a transmittance value of about 70 percent for the background in the darkest step. The plates were developed for three minutes in D–19 at 20 °C, and the transmittance values of the lines and the adjacent background were measured on a nonrecording microphotometer. The emulsion calibration was determined for each plate at 50 Å intervals by the two-step method [12].

Two sets of plates were exposed. One set was exposed with a solution containing 1 g/liter of iron (as ferric chloride), and the other with solutions containing 5 and 10 g/liter of iron. An exposure was also made on each set of plates with distilled water introduced into the arc to permit identification of interfering lines and bands. An additional exposure was also made on each set of plates of the continuous radiation from the anode of an 8-amp arc between graphite electrodes, arranged as described by Euler [13]. This permitted a correction to be made for the variation of response with wavelength of the emulsion and the spectrograph, using the temperature and emissivity data for the graphite anode given by Euler. The correction was interpolated through the *CN* band region. Finally an exposure was made on each set of plates of an iron arc for wavelength calibration.

### 5.2. Results

The effective excitation temperature of the discharge was found for each exposure with the three concentrations of iron, as described in section 3. The temperatures found were 5610, 5420, and 5510 °K, average 5510 °K. These data do not indicate any effect of the concentration of iron in the solution on the temperature of the discharge.

In the three exposures, the intensities of the Fe I lines increased by a larger factor than the increase in the concentration of iron in the solutions. For example, the line intensities increased by a factor of 2.9 when the concentration of iron in the solution was increased by a factor of 2.0, from 5 to 10 g/liter. This effect may be due to a shift in the ionization equilibrium, as has been observed [14] for the excitation of easily ionized elements in flames. If a significant portion of the electron concentration in the discharge is from ionization of iron, then the electron concentration will also increase with increased iron concentration. In accordance with the Saha equilibrium, this will result in a lesser degree of ionization as the total iron concentration is increased. Accordingly, the rise in the concentration of neutral iron atoms, and the intensity of the Fe I lines, can be expected to be greater than the increase in the total concentration of iron.

A total of 105 lines from the list of King and King [9] were observed in the three exposures. Several of the lines were not observed with a measurable intensity in all exposures, being either too light at the lowest concentration or too dark at the highest concentration. A few of the lines which could be observed on one set of plates could not be observed in the other set because they happened to come at the space between the two plates in the camera.

Relative *gj*-values for the 105 lines were calculated from the measured intensities using the relation *gf_r_*=I*_r_*λ^3^exp(*E/kT*), where *I_r_* and *f*_r_ are the relative intensities and oscillator strengths. The three sets of values were normalized to the same scale and an average *gf*-value was found for each line. To take into account the improvement in the precision of photometry with increasing line/background ratio, the *gf*-values from the three individual exposures were weighted approximately in proportion to the square root of the concentrations; the values obtained from the exposure with the most dilute solution were assigned a weight of 1, those from the exposure with the solution containing 5 g/liter of iron were assigned a weight of 2, and the values from the exposure with the most concentrated solution were assigned a weight of 3. The average deviation from the weighted mean of the values was also calculated for each line for which there were two or three measurements. The relative average deviation ranged from 0 to 15 percent, with an average of 4.2 percent. The relative *gf*-values were then normalized to the absolute scale of *gf* (3719.94 Å) = 0.288 [16].

Many of the lines observed in this study are subject to self-absorption, since the lower energy levels of the transitions are at or near the ground level. The extent of self-absorption in the gas-stabilized arc source was investigated by comparing the relative intensities obtained in the exposures with 5 and 10 g/liter of iron. For this purpose, the lines were divided into three groups, according to the degree of self-absorption found by Crosswhite [11] for these lines in a 1 amp d-c arc between iron electrodes. For 25 of these lines, Crosswhite found no significant self-absorption in the 1 amp arc; the ratio of the intensities at the two concentrations in the gas- stabilized arc, *I*_10_/*I*_5_, averaged 2.91, with a standard deviation of 0.24. For 31 of these lines, Crosswhite found less than 30 percent self-absorption; for these lines *I*_10_/*I*_5_=2.90±0.11. For 39 of the lines, Crosswhite found more than 30 percent self-absorption in the 1 amp arc; for these *I*_10_/*I*_5_=2.75±0.20. The difference in the ratio *I*_10_/*I*_5_ for the group of lines which showed more than 30 percent self-absorption in the 1 amp arc and the other two groups of lines is statistically significant. While the effect of self- absorption is detectable, it has not been a serious cause of error in our work. The ratio *I*_10_/*I*_5_ changed by 5.5 percent between the lines which were found by Cross white to be not self-absorbed and those that showed more than 30 percent self absorption in the 1 amp arc; this difference is comparable to the photometric error.

Table 1 lists the *gf*-values found in this study. The wavelengths and energy levels are taken from the tables of Meggers, Corliss, and Scribner [15]. The *gf*-values found for these lines by King and King [9], by Crosswhite [11], and by Corliss and Bozman [5] are also listed. (Crosswhite’s tables list *e*-values which are related to the *gf*-values by a wavelength-dependent conversion factor. Crosswhite states that multiplication of the *e*-values by the conversion factor will give the *f*-values. Actually, this operation gives the *gf*-values.) The *gf*-values given by the Kings and by Crosswhite are also placed on the absolute scale based on *gf*(3719.94 Å)=0.288 [16]. Crosswhite’s values had been normalized to an earlier [17] absolute measurement of the same Fe I line which was lower by about a factor of three.

### 5.3. Discussion

**Figure 3:**
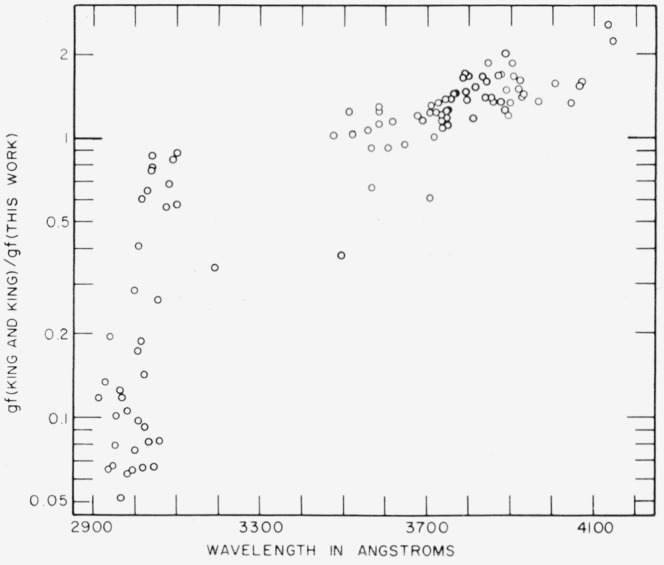
Comparison of *gf*-values from this study with values given by King and King [9], showing wavelength-dependent difference.

Figure 3 shows a comparison between the results of this work and the data given by King and King [9]. The ratio of the *gf*-values from the two studies is plotted as a function of the wavelength of each line. The wavelength-dependent error in the values of King and King, first noted by Crosswhite [11], is shown clearly. The points fall low at short wavelengths and they also show increased scatter compared to the points at the longer wavelengths. These data tend to confirm Crosswhite’s deduction that the values obtained by the Kings are low at shorter wavelengths apparently because of scattered light in the spectrograph.

**Figure 4:**
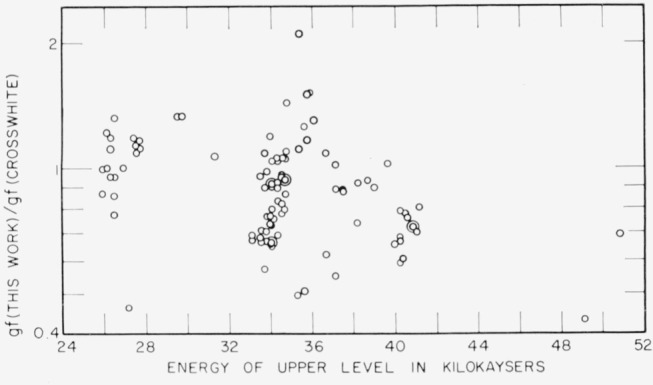
Comparison of *gf*-values from this study with values given by Crosswhite [11], showing energy-dependent difference.

Figure 4 is a comparison of the data obtained in this study and the results given by Crosswhite [11]. The ratio of the *gf*-values is plotted as a function of the energy of the upper state of the transition. Although the points are scattered, there appears to be a downward trend of the ratio of the *gf*-values with the excitation energy of the line. This energy- dependent difference reflects a systematic difference between the data of Crosswhite and the values given by Corliss and Bozman [5]. The data listed by Corliss and Bozman are derived from the measurements by Meggers, Corliss, and Scribner [15] of line intensities in a copper arc. Corliss [18] found the temperature of this arc to be 5100 ±110 °K, and this temperature was used by Corliss and Bozman in the calculation of the *gf*-values. With the *gf*-values given by Crosswhite and the line intensities listed by Meggers, Corliss, and Scribner, a temperature of 4750 °K is found for the copper arc. There is, therefore, an energy-dependent bias between the *gf*-values obtained by Crosswhite and those given by Corliss and Bozman.

**Figure 5:**
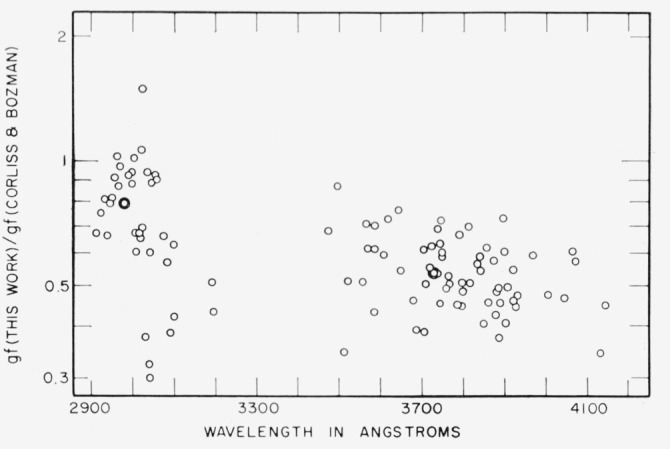
Comparison of *gf*-values from this study with values given by Corliss and Bozman [5], showing wavelength-dependent difference.

**Figure 6:**
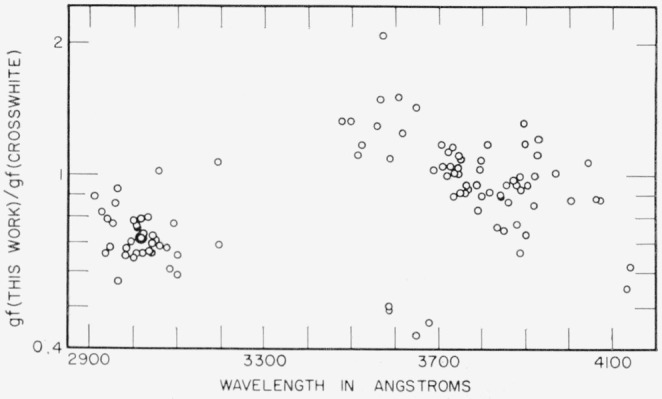
Comparison of *gf*-values from this study with values given by Crosswhite [11], showing wavelength- dependent difference.

The present measurements are related to those of Corliss and Bozman in such a way that there can be no systematic differences that are dependent on the excitation energies of the lines. However, a wavelength-dependent difference is possible. Figure 5 is a comparison of the data of this study with those of Corliss and Bozman, plotted as a function of wavelength. These appears to be a slight trend of the ratio of the *gf*-values with wavelength. The same comparison when made between the results of this work and those of Crosswhite shows an indication of a trend in the opposite direction (fig. 6). Figure 5 also shows how the absolute scale of Corliss and Bozman differs from the one adopted in this work.

The *gf*-values resulting from the work described here have a random error of 4.2 percent (see sec. 5.2.) Corliss and Bozman estimate the precision of their *gf*-values at 30 to 35 percent in the range of excitation energies of these lines, and their data agree with the values of the present study within this factor, aside from the difference in the absolute scale. Crosswhite does not give an estimate of the precision of his values, but an indication of the random errors involved in his measurements can be obtained by comparison of the values obtained by him from measurements with different sources. Table 2 lists the results of such a comparison. Good agreement between two sets of results is indicated when the ratio of the *gf*-values is near unity and the standard deviation of the ratio is small. Good agreement according to this criterion is found in some instances, but in other cases the average of the ratios is far from unity or the standard deviation is quite large. Overall, the relative standard deviation is 44 percent. The *gf*-values listed in table 1, and compared with the results of this study in figures 4 and 6 have been selected by Crosswhite as being probably the most reliable, so that the random errors may not be as large as the 44 percent relative standard deviation found by the internal comparison. However, it is not unreasonable to assume that much of the scatter of the points in figures 4 and 6 represents random errors in the measurements made by Crosswhite.

## 6. Conclusions

The results of the present study indicate that the gas-stabilized arc source should be useful for the determination of transition probabilities. The source appears to exhibit local thermal equilibrium but it does have a large radial temperature gradient. The spectral intensities emitted by the arc are remarkably constant even though the arc is not completely stable in position. The absolute concentrations of the elements in the discharge are not known, so that only relative transition probabilities may be determined directly. However, one experiment indicated that when a solution containing a mixture of elements is introduced into the arc the relative concentrations of the elements in the plasma are approximately the same as in the solution. It may therefore be possible to determine absolute transition probabilities by means of measurements with mixed solutions, employing known absolute values for one element to place relative values for another element on an absolute scale. The effects of self-absorption are not large in the gas-stabilized arc source, and they can be studied by varying the composition of the solution that is introduced into the discharge.

The gas-stabilized arc source has been applied to the redetermination of oscillator strengths for 105 lines in the first spectrum of iron. Systematic differences have been found between the results of this study and those of King and King [9], Crosswhite [11], and Corliss and Bozman [5]. The new data do not make it possible to select any one set of *gf*-values as being more accurate than the others, except that a wavelength-dependent error in the data of King and King, first noted by Crosswhite, has been confirmed. The new measurements are believed to have smaller random errors than any of the previous values.

## Figures and Tables

**Table 1:** Excitation energies and oscillator strengths for lines of Fe *I*

Wavelength^a^	Energy levels a Kaysers (em^−1^)		gf-values			
	Lower	Upper	This work	Corliss & Bozman	Cross-white b	King & King^b^
						
2912.16	0	34329	0.0459	0.068	0.0513	0.0054
2929.01	416	34547	.0548	.073	.0665	.0073
2936.90	0	34040	.213	.26	.322	.014
2941.34	704	34692	.0331	.050	.0417	.0065
2947.88	416	34329	.222	.28	.324	.015
2953.94	704	34547	.188	.23	.243	.015
2957.36	888	34692	.119	.13	.138	.012
2965.26	978	34692	.0728	.071	.0782	.0092
2966.90	0	33695	.446	.51	.780	.023
2970.10	888	34547	.127	.13	.011	.015
2981.45	416	33947	.0722	.091	.11	.0076
2983.57	0	33507	.318	.40	.469	.020
2994.43	416	33802	.354	.38	.503	.023
2999.51	6928	40257	.669	.71	.85	.19
3000.95	704	34017	.328	.37	.509	.025
3007.28	404	33947	.0344	.033	.045	.0060
3008.14	888	34122	.194	.32	.294	.019
3009.57	7377	40594	.410	.61	.54	.17
3017.63	888	34017	.0462	.069	.0584	.0087
3018.98	7728	40842	.280	.43	.39	.17
3021.07	416	33507	.512	.48	.773	.034
0024.03	888	33947	.0607	.088	.083	.0087
3025.84	978	34017	.195	.13	.268	.018
3031.64	8155	41131	.184	.49	.23	.12
3037.39	888	33802	.292	.31	.437	.024
3040.43	7377	40257	.113	.35	.17	.087
3042.02	8155	41018	.0905	.15	.13	.071
3042.66	7986	40842	.138	.46	.19	.12
3047.60	704	33507	.358	.40	.505	.024
3057.45	6928	39626	1.02	1.1	1	.27
3059.09	416	33096	0.253	0.28	0.368	0.021
3075.72	7728	40231	.476	.72	.7	.27
3083.74	7986	40405	.303	.53	.5	.21
3091.58	8155	40491	.178	.46	.23	.15
3100.30	7986	40231	.236	.56	.4	.21
3100.67	7728	39970	.327	.52	.5	.19
3193.23	0	31307	.0107	.021	.01	.0037
3196.93	19562	50833	3.67	8.5	5.3	c.0022
3476.70	978	29733	0.0341	0.050	0.0256	.035
3497.84	888	29469	.0314	.036	.0236	.012
3513.82	6928	35379	.111	.32	.10	.14
3521.26	7377	35768	.164	.32	.14	.17
3558.52	7986	36079	.338	.66	.26	.36
3565.38	7728	35768	1.06	1.5	.71	.98
3570.10	7377	35379	2.09	3.4	1.0	1.4
3585.32	7728	35612	0.231	0.33	0.46	0.26
3585.71	7377	35257	.108	.25	.22	.14
3586.99	7986	35856	.240	.39	.22	.30
3608.86	8155	35856	1.19	2.0	.79	1.1
3618.77	7986	35612	1.38	1.9	1.1	1.6
3647.84	7377	34782	0.918	1.2	0.64	0.87
3649.51	21716	49109	3.31	6.1	7.7	c.0015
3679.92	0	27167	0.0272	0.059	0.0226	.033
3687.46	6928	34040	.199	.51	.193	.23
3705.57	416	27395	.0482	.079	.0409	.060
3707.82	704	27666	.0116	.030	------	.0071
3709.25	7377	34329	.298	.59	.285	.39
3719.94	0	26875	.288	.52	.288	.29
3722.56	704	27560	.0525	.084	.0464	.065
3727.62	7728	34547	.307	.57	.292	.41
3733.32	888	27666	0.0410	0.076	0.0354	0.050
3734.87	6928	33695	2.25	4.2	2.51	2.6
3737.13	416	27167	0.220	0.32	0.215	0.24
3743.36	7986	34692	.217	.48	.208	.30
3745.56	704	27395	.152	.24	.15	.18
3745.90	978	27666	0.0476	0.066	0.043	0.060
3748.26	888	27560	.0902	.15	.0829	.10
3749.49	7377	34040	1.59	2.7	1.74	2.0
3758.24	7728	34329	1.08	2.2	1.18	1.5
3763.79	7986	34547	0.686	1.3	0.724	0.98
3767.19	8155	34692	.489	0.97	.526	.71
3787.88	8155	34547	.162	.36	.170	.27
3790.10	7986	34363	.0300	.045	.036	.051
3795.00	7986	34329	.219	.49	.213	.32
3798.51	7377	33695	.102	.20	.0941	.14
3799.55	7728	34040	.160	.33	.178	.27
3812.96	7728	33947	.153	.22	.13	.18
3815.84	11976	38175	1.83	3.6	2.0	2.8
3834.22	7728	33802	0.485	0.86	0.637	0.81
3840.44	7986	34017	.335	.57	.371	.47
3841.05	12969	38996	1.25	2.3	1.4	2.0
3849.97	8155	34122	0.150	0.37	0.20	0.28
3856.37	416	26340	.0433	.070	.0454	.060
3859.91	0	25900	.141	.31	.162	.19
3872.50	7986	33802	.132	.23	.135	.22
3878.02	7728	33507	.136	.32	.143	.23
3878.58	704	26479	.0358	.074	.0464	.048
3886.28	416	26140	.0689	.14	.0690	.087
0887.05	7377	33096	.0748	.20	.112	.15
3888.52	12969	38678	.436	.96	.47	.65
3895.66	888	26550	.0233	.032	.0176	.028
3899.71	704	26340	.0284	.047	.0241	.038
3902.95	12561	38175	.528	1.3	.72	.98
3906.48	888	26479	.00597	0.012	.00628	.010
3920.26	978	26479	.0175	.032	.0204	.026
3922.91	416	25900	.0193	.042	.0194	.031
3927.92	888	26340	.0271	.061	.0241	.038
3930.30	704	26140	.0293	.062	.0241	.042
3969.26	11976	37163	.594	1.0	.59	.81
4005.25	12561	37521	.413	0.87	.47	.65
4045.82	11976	36686	2.48	5.3	2.3	3.3
4063.60	12561	37163	1.51	2.5	1.7	2.3
4071.74	12969	37521	1.32	2.3	1.5	2.1
4132.06	12969	37163	0.237	0.69	0.40	0.60
4143.87	12561	36686	.341	.76	.55	.76

a Wavelength and energy level values taken from Meggers, Corliss and Scribner [15].

b Relative values given by King and King [9] have been normalized to an absolute scale of *gf* (3719.94Å)=0.288 [16]. Absolute values given by Crosswhite [11] have been recalculated to the same absolute scale. See text for details.

c The energy levels assigned to these lines in the work of King and King [9] are considerably different from those assigned in the tables of Meggers, Corliss and Scribner [15].

**Table 2:** Comparison of *gf*-values measured with different sources Data of Crosswhite [11].

Sources*	a/d	a/e	a/f	a/h	d/e	d/f	d/h	e/f	e/h	f/h
Average ratio of *gf*-values	1.86	0.90	0.82	0.96	1.15	1.00	0.65	0.99	0.97	1.01
Standard deviation of ratio	0.94	.32	.27	.73	0.26	0.096	.34	.22	.46	0.094
Number of lines compared	21	22	22	51	11	11	26	24	32	32

* The letters correspond to *gf*-values measured with the following sources:

a–1 amp d-c arc; d–flame in absorption; e–electrodeless discharge; f–flame in emission; h–hollow cathode.

## Footnotes

1 Figures in brackets indicate the literature references at the end of this paper.
